# Spanish Spelt Wheat: From an Endangered Genetic Resource to a Trendy Crop

**DOI:** 10.3390/plants10122748

**Published:** 2021-12-13

**Authors:** Juan B. Alvarez

**Affiliations:** Edificio Gregor Mendel, Departamento de Genética, Campus de Rabanales, Escuela Técnica Superior de Ingeniería Agronómica y de Montes, Universidad de Córdoba, CeiA3, ES-14071 Córdoba, Spain; jb.alvarez@uco.es

**Keywords:** agrobiodiversity, ancient wheat, genetic resources, neglected crops

## Abstract

Spelt wheat (*Triticum aestivum* L. ssp. *spelta* Thell.) is an ancient wheat that was widely cultivated in the past. This species derived from a cross between emmer wheat (*T. turgidum* spp. *dicoccum* Schrank em. Thell.) and *Aegilops tauschii* Coss. Its main origin was in the Fertile Crescent (Near East), with a secondary center of origin in Europe due to a second hybridization event between emmer and hexaploid wheat. This species has been neglected in most of Europe; however, the desire for more natural foods has driven a revival in interest. Iberian spelt is classified as a geographical group differing to the rest of European spelt. In this review, the particularities, genetic diversity and current situation of Spanish spelt, mainly for quality traits, are discussed.

## 1. Introduction

The area known as the Fertile Crescent (Near East) has been widely suggested by numerous authors as the origin of wheat [1]. Under the name of wheat is possible to find numerous species and subspecies from the genus *Triticum*, grouped in three ploidy levels: diploid (2n = 2× = 14, A^m^A^m^), tetraploid (2n = 4× = 28, A^u^A^u^BB) and hexaploid (2n = 6× = 42, A^u^A^u^BBDD). Wild ancestors have been found for the diploid and tetraploid species, which were domesticated by hunter-gathering societies during their evolution to farming societies [2]. The first archaeological traces of wheat have been identified as from around 10,000 BP (pre-pottery Neolithic A), being mainly diploid and tetraploid species. In contrast, all hexaploid wheats are domesticated, having their origin in natural crossing between emmer wheat (*Triticum turgidum* spp. *dicoccum* Schrank em. Thell., 2n = 4× = 28, A^u^A^u^BB), the first domesticated tetraploid wheat, and a wild grass (*Aegilops tauschii* ssp. *strangulata* Coss., 2n = 2× = 14, DD), which could have at least two separate origins: one in Iran and another in Turkey or northern Syria [3]. This event was recreated by McFadden and Sears [4], obtaining plants with high similitude to the ancient wheat known as spelt (*T. aestivum* L. ssp. *spelta* Thell., 2n = 6× = 42, A^u^A^u^BBDD), and considered to be the ancestor of common wheat (*T. aestivum* L. ssp. *aestivum*, 2n = 6× = 42, A^u^A^u^BBDD).

A third origin of spelt outside the Fertile Crescent has also been suggested [5]; European spelt could be derived from a secondary hybridization between emmer wheat and hexaploid wheat, probably club wheat (*T. aestivum* L. ssp. *compactum* Host em. Mackey) [6,7,8,9]. In addition to this, European spelt could be classified into two eco-geographical groups: Iberian (pol. *ibericum* Flaskb.) and Bavarian (pol. *bavaricum* Vav.) groups, being the first originated from Asia and show great differences to the spelt from Central Europe that likely originated following secondary hybridization [9,10]

**Figure 1:**
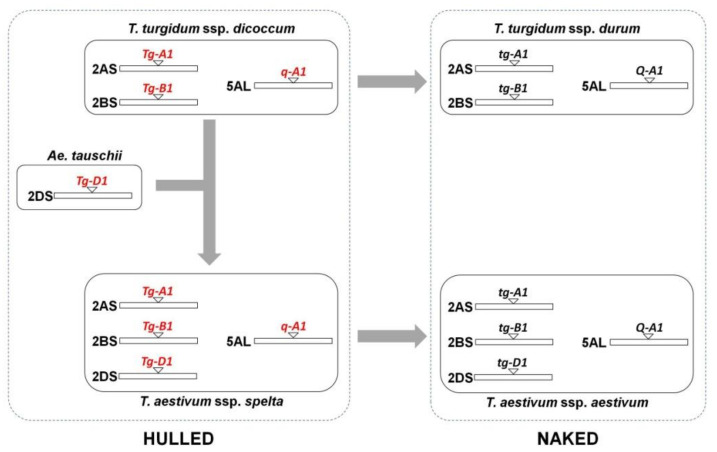
Evolution of genes related to the domestication in *Triticum* species. The free-threshing character is controlled by the *Q* gene and the tenacious glume by the *Tg* gene.

Some morphological characteristics of spelt clearly differ from common wheat, including non-free threshability, brittle rachis, long spikes and hulled grains [11,12,13,14,15], which mainly arose from the expression of two loci: *Q* and *Tg* genes. The *Q* gene encodes a transcription factor (*APTETALA2* family) related to these characteristics [16,17]. The first studies found the presence of a recessive allele (*q*) in common wheat that is key in the morphological differentiation between spelt and common wheat. This locus is on the long arm of chromosome 5A in polyploid wheat [6,18]. The other gene, *Tg*, is related to glume tenacity (i.e., hulled grain) and located on the short arm of chromosome 2 of each wheat subgenome. The first gene evaluated was *Tg-D1*, found by Kerber and Rowland [19], in populations derived from crosses between durum wheat and *Ae. tauschii*, and the observed non-free-threshing trait was due to the presence of a dominant allele (*Tg-D1*) from *Ae. tauschii*. This suggested that this gene was epistatic to the *Q* gene. Later, orthologous genes were detected in chromosome 2BS (*Tg-B1*) by Simonetti et al. [20], using crosses between durum and emmer wheat, and in chromosome 2AS (*Tg-A1*) by Dvorak et al. [21] in spelt. Data suggested that mutations of *Tg-A1*, *Tg-B1* and *Q* present in emmer wheat were responsible for the free-threshing trait in modern durum wheat [22]. This process was later carried out in hexaploid wheat (spelt), including the mutation on *Tg-D1* (Figure 1).

In recent times, the presence of these allelic variants in any modern material has been sufficient for considering as spelt. This has led to two different spelt types: traditional/pure and modern/synthetic [23]. The latter are derived from crosses between semi-dwarf common wheat and traditional spelt, which show spikes with speltoid morphology and tenacious glumes. In contrast, traditional spelts are obtained by selection among old spelt landraces. Although both options are completely feasible, the differences between them should be made clear for free choices by consumers.

## 2. History of Spelt Cultivation and Use

As mentioned above, spelt may have been synthesized in at least two different locations within the Fertile Crescent. One of the main problems in clearly determining the presence of specific wheat species is the difficulty in identifying seeds using morphological features in any archaeological site [24]. This has been recently mitigated by the application of ancient DNA techniques to identify vegetal material from archaeological sites [25,26,27], which has shown a bright light on wheat evolution.

Another difficulty is the identification of this species in ancient texts, because the actual name, spelt, is a Latin word with Germanic origin that first appears in an Imperial edict on prices by the imperator Diocletian in the 4th century (“*Edictum de maximis pretiis rerum venalium*”, 301 AD). Previously, this species may have been referred to with an epithet similar to emmer and these names could have been confused in translations from Greek or Latin to vernacular languages. In some Latin texts, such as “*Naturalis Historia*” (Natural History) by Pliny the Elder, this species is named *scandŭla*, from which is derived the Spanish term for this species (*escanda*), probably due to the tendency of this species to have a hanging spike. Some evidence suggests this was probably also known as *far*, a name shared by the three hulled wheat species: einkorn (*T. monococcum* L. ssp. *monococcum*, 2n = 2× = 14, A^m^A^m^), emmer and spelt.

In any case, the historical records show that spelt has been an important staple food since its remote origin [28]. During all this time, spelt was generally consumed as bread, because its flour is better for bread-making than the other ancient wheats einkorn and emmer. However, its expansion to Europe was in the later Neolithic (2500–1700 BC), extending into regions of eastern Germany, Poland and Jutland [29]. However, it was during the Bronze Age that its cultivation spread widely in Northern Europe, replacing emmer as the main wheat species during the Iron Age (750–15 BC). In this last period, it appeared in southern Britain and northern Spain [30,31]. In Spain, references to its cultivation can be found in Christian texts from the Middle Ages such as the “*Cronicon Albendense*” dated in 883, where it is described as Asturian *fisga*, and in Arab agronomic treatises such as the “*Agriculture’s Book*” of Ibn Al Awam. During this historical phase its decline began, with progressive substitution by common wheat due to its high grain yield and better bread-making properties. For a long time, spelt was used to pay rent, suggesting that it was considered a valuable material. When rents began to be paid in cash, this species lost its value and began to decline.

This neglect was intense and, in the 20th century, its cultivation was restricted to a few regions of Germany, Switzerland, Belgium, Italy and Spain with a much-reduced harvested area [32]. For example, in Spain, especially after the Spanish Civil War (1936–39), the erosion and neglect of spelt was notable, especially in the 1960s and 1970s when the cultivated area was less than 15 ha distributed in small parcels destined for home consumption [32]. At present, spelt is grown mainly in Germany (≈100,000 ha), Austria (≈13,000 ha) or Switzerland (≈5500 ha); being the cultivated area in Spain sensitive lower, where it is mostly grown in organic farming. The trend in Spain due to the growing interest in this crop is the gradual increase in this cultivated area, although still far from other European countries.

## 3. The Past and Present Diversity of Spelt in the Iberian Peninsula

In the 19th century, the Spanish botanists Lagasca and Clemente cataloged at least seven botanical spelt varieties (Table 1): two of the *mutica* group (vars. *albivelutinum* Körn. and *album* (Alef.) Körn.) and five from the *aristata* group (vars. *arduinii* (Mazz.) Körn., *caeruleum* (Alef.) Körn., *duhamelianum* (Mazz.) Koern., *rubrivelutinum* Körn. and *vulpinum* (Alef.) Körn.) [33]. This suggested a low variability of this species compared to others included in this unpublished herbarium (named “*Ceres Hispanica*”) stored in the Royal Botanic Garden of Madrid, Spain.

Fortunately, many of these materials were conserved in germplasm banks, which allowed recovery of this variability for use in cultivation of traditional spelt. The main collections of Spanish spelt germplasm are in two germplasm banks: the USDA-National Small Grain Collection (Abeerden, Idaho, USA) contains up to 405 accessions of Spanish origin and the collection of the CRF-INIA (Centro de Recursos Fitogenético, Alcalá de Hernares, Spain) has 115 accessions. Other germplasm banks have multiplications of these materials.

The first collection includes a set of 333 accessions collected in one Spanish region (Asturias in northern Spain), where spelt is still cultivated, mainly by small farmers for self-consumption. This set has a curious history because, according to data of the Swiss Federal Research Station for Agroecology and Agriculture, which originally collected these accessions, these were collected in the 1930s—in the years immediately before the Spanish Civil War or even during this fratricidal conflict—and were stored in this Swiss institution in 1939 [Dr. G. Kleijer 2004, pers. commun.].

**Figure 2:**
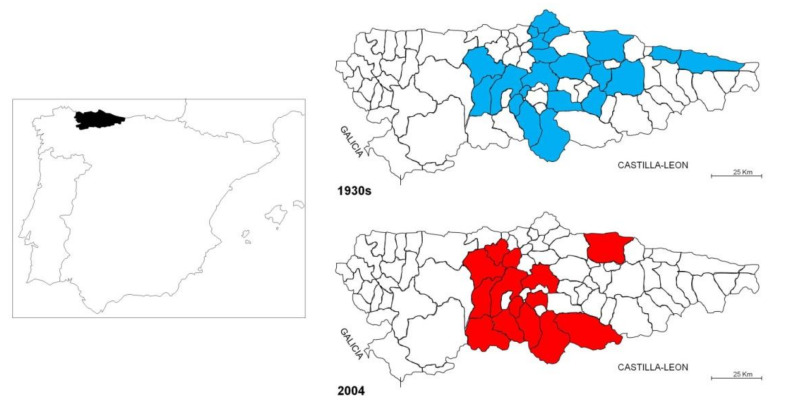
Situation of Asturias (Northern of Spain) *concejos* where spelt accessions were collected as from the collecting missions of 1930s and in 2004.

During the Swiss expedition of the 1930s (Figure 2), spelt was collected in 50 sites distributed in 23 *concejos* (local units or municipalities in Asturias). This indicated a clear decrease in the cultivation zones compared to the beginning of the 20th century as recorded by Alvargonzalez [34], who wrote that spelt was cultivated in 37 *concejos* of out 78 from Asturias with a production around 960 metric tons. In 2004 (Figure 2), a new collecting mission was carried out, which followed the approximate route of the Swiss expedition [35,36]. In this case, the zones where it is still maintained, the cultivation of spelt were identified by previous phone calls to the all *concejos* from Asturias. The after expedition permitted to collected spelt in 31 locations distributed in 13 *concejos* (Figure 2). Only seven out of these 23 *concejos* were common to both expeditions, indicating a great decline in spelt cultivation. In the collected parcels, spelt appears in general as pure crop without mix with other cereals. Only in two parcels was detected together with emmer wheat, which the most farmers considerate as a weed.

Another possible risk factor of genetic erosion observed during the 2004 collecting mission was the small size of the cultivation area, together with the low number of farmers, in each location. In most cases, spelt was grown in small areas not greater than 200 m^2^ and one unique farmer in each case. Additionally, there was a high average age of spelt growers, who were mainly women. These materials were grown for self-consumption using archaic agronomical techniques [35], which include the hand-harvest using *mesorias* (a rudimentary instruments made by two sticks joined by a short piece of leather) or the hulling with *rabil* (a species of air mil). Only in two *concejos* were larger farms found; in both cases, these farms belonged to small cereals companies dedicated to cultivation of spelt on a larger scale. In these cases, a greater heterogeneity was observed within the plots, probably due to the fact that these materials were generated from mixtures obtained from several small farmers throughout Asturias. Nowadays, great part of the spelt flour from Asturias is commercialized by these companies.

Both the old (1930s) and new collections were evaluated for morphological traits with the aims of identifying the botanical varieties present in each collection [36,37], and comparing these with the data of Lagasca and Clemente [33]. On the basis of the chosen morphological discriminators, six botanical varieties were identified in the collection from the 1930s, which represents a little erosion with respect to the botanical varieties detected in the 19th century [33]. Only one of these was awnless, having red and glabrous glumes (var. *duhamelianum*). The other five were all awned: one had blue pubescent awns (var. *caeruleum*), one had white glabrous glumes (var. *arduinii*), one had white pubescent glumes (var. *albivelutinum*), one had red glabrous glumes (var. *vulpinum*) and one had red pubescent glumes (var. *rubrivelutinum*) [37].

**Figure 3:**
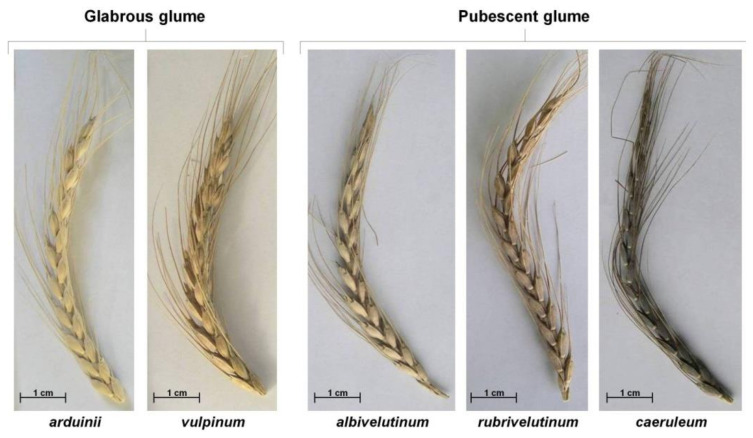
Botanical varieties cataloged by Lagasca and Clemente [33] detected in Asturias (Northern of Spain) during the collecting mission of 2004.

The materials collected in the summer of 2004 were also morphologically evaluated [36] and classified according to the botanical varieties indicated in the “*Ceres Hispanica*” herbarium [33]. No variety of the *mutica* group was found in the last collecting mission [36], but five of seven botanical varieties described by Lagasca and Clemente (vars. *albivelutinum*, *arduinii*, *caeruleum*, *rubrivelutinum* and *vulpinum*) were detected (Figure 3). This indicates that the spelt currently grown in Asturias has variability levels similar to the spelt collected in the 1930s. On the other hand, the frequency of each botanical variety was related to its acceptance by farmers. In general, they prefer spikes with white glumes (vars. *albivelutinum* and *arduinii*,), this type of spike being present in half of the collected accessions. Other colors such as blue spikes (var. *caeruleum*) are, clearly, less accepted, and their tendency is to disappear.

The presence of materials not described in “*Ceres Hispanica*,” which may have been introduced in recent times from Central Europe, should be noted [35]. Two genotype groups with yellow spikes, one with glabrous glumes and another with pubescent ones, were detected; none of them was previously described in Spanish materials. These materials, although they are not possible autochthons, showed typical characteristics of traditional spelt. In contrast, some materials derived from modern spelt (i.e., crossed with common wheat) with short plant height and awnless spikes were detected, but with few examples due to poor adaptation to the edaphic and climatic conditions of Asturias [35]. These are characterized by poor soils with a high slope, and a high rainfall that favors long cycles. Typically in Asturias, spelt is sown in November-December and harvested in June-July.

## 4. Spelt as a Source of Useful Genes for Breeding and as a Crop

The neglect of spelt as a crop has not meant its neglect as an interesting species for research studies on the origin and domestication of common wheat [38,39], studies that with more or less controversy have continued [5,21]. In addition, this species was successfully evaluated as a source of resistance genes for some wheat diseases. The first studies were carried out on resistance to rusts, mainly wheat yellow rust (*Puccinia striiformis* West.), and detected some interesting genes (*Yr5* or *Yr10*) that were transferred to common wheat [40]. Other genes for resistance to leaf rust (*P. recondita* f. sp. *tritici*), such as *Lr44*, *Lr65* or *Lr71* [41,42,43,44,45,46], were also detected in spelt accessions, and also for stem rust (*P. graminis* f. sp. *tritici*) [47]. Furthermore, sources of resistance to powdery mildew (*Erysiphe graminis* f. sp. *tritici*) [41,48] or Septoria tritici blotch (*Zymoseptoria tritici*) [49,50] were associated with spelt. However, the presence of other characters considered undesirable, mainly brittle rachis, tall plants or hulled grains, limit its use for breeding other agronomical traits.

Nevertheless, during the last decade of the 20th century, the search for natural foods permitted the revival of numerous neglected crops, including spelt [51,52,53,54,55]. This renewed interest in spelt is mainly linked to its use as food, since its grain yield is lower than that of other crops such as common wheat [56]. For farmers, the profitability of this ancient crop would be associated with the high price of the grain and not with its potential grain yield. For this reason, this crop has been more successful in organic farming, where inputs are significantly lower, and the differences for grain yield face to common wheat are notability lower according with some studies carried out in several European zones [57,58].

Evaluation of materials stored in germplasm banks is the key to recovering this crop for human consumption. In this respect, the technological parameters usually used for quality analysis in common wheat have been evaluated in these old materials [59,60,61,62,63]. This has two aims: to identify possible quality traits specific to spelt, and to determine the most adequate use of each one of these materials. Although spelt has generally been designated for making bread, its technological characteristics indicate that it could be used for making other food products such as biscuits, cakes, pasta and breakfast cereals [64]. Although evaluating old wheats using modern parameters should be carried out with caution, spelt has good baking quality [60,61], detaching the color and imparts a distinctive taste to products made with its flour [65]. This trait is very important because when these traditional products are made with modern wheat flour, the organoleptic quality, mainly smell, taste and texture, is clearly lower and can result in rejection by consumers. For this reason, the revival of ancient wheats has gone hand-in-hand with the revival of traditional foods.

**Figure 4:**
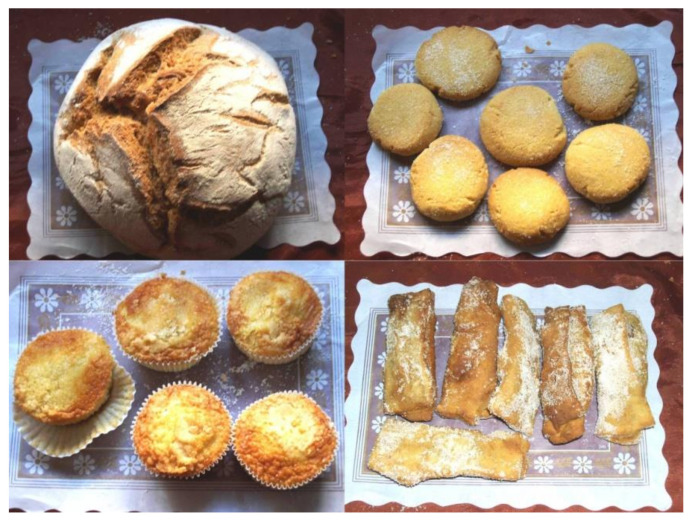
Traditional products made with spelt in Asturias. Left to right, top to bottom: bread, biscuits, cupcakes and pancakes (*freixuelos*).

Spain has also been affected by this global movement, and the recovery of the autochthon materials began in the last decade of the 20th century [32], these type of materials being the most used for spelt cultivation in Spain at now. This has revived consumption of some traditional products that use spelt as raw material (Figure 4). In addition to bread, cookies or cupcakes, the traditional Asturian cuisine has used the spelt flour in many elaborations: *panchón* (crumbled bread mixed with butter and sugar), *fórmigos* (bread crumbs, eggs, milk and sugar fried and sprinkled with wine), *freixuelos* (pancake) and *faricos* (baby food). However, although now these products are considerate gourmet food, a few years ago their consumption was very local and only was made in small bakeries named «*obradores*». Nowadays, the spelt flour is easy to find in the market and it is used to elaborate many products, although the percentage of spelt flour in most of these products is relatively low.

In parallel to this search for natural foods, worries concerning the nutritional quality of modern products has aroused interest in the development of materials with higher levels of micronutrients, such as zinc, iron or selenium needed for correct nutrition [66,67]. Diverse studies, in some cases with discordant results and conclusions, have suggested differences in grain mineral composition between spelt and common wheat [68,69,70,71], showing spelt higher contents of zinc or iron. One of the main causes of this controversy is likely due to differences in the milling of spelt compared to common wheat, which for spelt generates flour with higher amounts of bran and the aleurone layer [72]. However, recent studies showed that some of these traits, mainly zinc and iron content, can be transferred from spelt to common wheat by crosses with donor lines of spelt [73,74,75,76]. This would permit the development of biofortified wheat (common and spelt) with good technological qualities [76].

Another important issue with cereal-based foods is sensitivity to gluten or other grain components. The two main pathologies associated with gluten proteins are celiac disease and non-celiac gluten sensitivity, which affects up to 6–8% of the worldwide population [77,78]. Part of the resurgence in use of spelt and other ancient wheats is driven by the false belief that these materials can be consumed by celiac patients. However, some studies have shown that the proteins most responsible for this reactivity in hexaploid wheat are the gliadins derived from the *Ae. tauschii* genome [79]. Due to its origin, these proteins are present in spelt and so in this regard there is no difference between spelt and common wheat. Thus, consumption of spelt by celiac patients should be ruled out.

## 5. Variation for Quality Parameters

Due to the strong recession that the crop suffered during the second half of the 20th century in Spain, the materials available to farmers may lack variation or be the result of mixing processes from different sources as already mentioned. In new situation, where diversification of uses of spelt flour is sought, this may be highly conditioned, and the potential of the crop as a food may be masked by this heterogeneity. From a technological quality point of view, it is necessary to study the possible variation both in the material in use and in the one stored in germplasm banks, and thus evaluate its suitability for the different uses, present or future, of spelt flour.

There are three main constituents of wheat grains that are related to their technological quality: starch, storage proteins and puroindolines. These components are present in the grain endosperm and constitute most of the flour or semolina. Starch is the main component of grains, being formed by two D-glucose polymers: amylose and amylopectin. Numerous enzymes are involved in its synthesis, and their presence/absence or functionality have been evaluated [80]. Starch is stored as granules within a protein matrix formed by seed storage proteins; both components are involved in the germination process, proving energy and amino acids for the new plant. The denaturation of these seed storage proteins by mechanical mixing generates one macroscopic structure named gluten, which is the key to baking and pasting processes [81]. The puroindolines affect the grain texture, which influences grain milling and indirectly affects water absorption of flour during the dough process [82].

**Figure 5:**
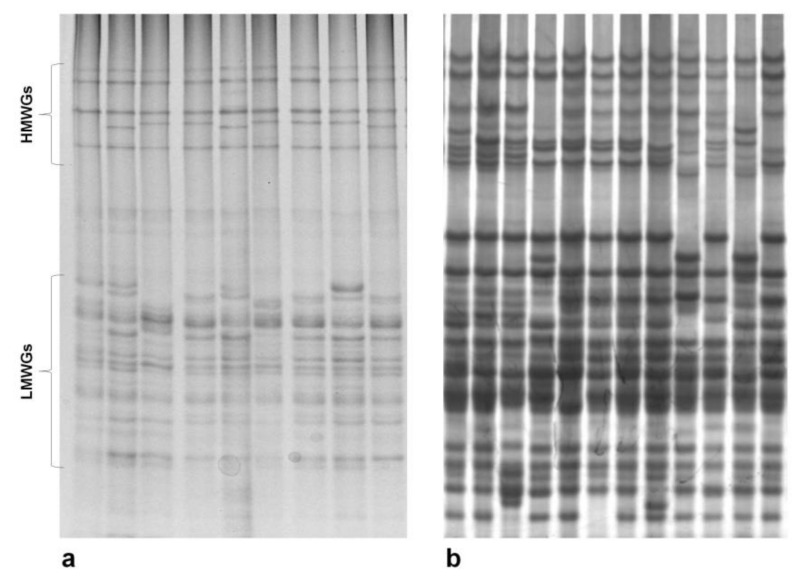
Electrophoretic separation of seed storage proteins from spelt. (**a**) glutenins and (**b**) gliadins. HMWGs: high-molecular-weight glutenin subunits and LMWGs: low-molecular-weight glutenin subunits.

These traits have been evaluated in Spanish spelt in recent years. The first studies were carried out on seed storage proteins, using one selection derived from the important spelt collection stored in the USDA-NSGC [83]. The complete evaluation of this collection that including the 333 accessions collected in 1930s (see Section 3) was then carried out for seed storage proteins: glutenins and gliadins (Figure 5) [84,85,86,87].

In studies of the high-molecular-weight glutenin subunits (HMWGs) encoded by the *Glu-1* loci [88], data showed that certain diversity remained in these materials, although with a high danger of erosion by genetic drift. Up to 19 allelic variants (three alleles at the *Glu-A1* locus, seven at *Glu-B1* and nine at *Glu-D1*) were found in the evaluated accessions, which were combined in 25 different patterns [84]. One of these patterns (subunits 1, 13 + 16 and 2 + 12) was very frequent, being present in 67.74% of these accessions [84]; however, 11 were found in only one accession. These materials present in high frequency the subunits 13 + 16 (allele *Glu-B1f*), associated with good bread-making quality, which is rare or appear with low frequency in common wheat [89,90,91,92]; although this was detected in old landraces of common wheat from the south of Spain [93]. These frequency variations suggest that some of these alleles could miss due to genetic drift processes. The data obtained by Caballero et al. [94] using sodium dodecyl sulfate-sedimentation test [95] showed that this allele (*Glu-B1f*), together with two of its variants (13* + 16 *Glu-B1ba* and 13 + 18 *Glu-B1at*), had the highest values (14.5, 13.5 and 13.4 mL, respectively). However, it was the presence of the allele *Glu-D1an* (2 + 12*) that showed the highest value for this test (17.3 mL). Unfortunately, this last allele together with four of *Glu-B1* locus (*Glu-B1an*−*6 + null-*, *Glu-B1e −20x + 20y-*, *Glu-B1bb −6 + 18′-* and Glu-B1ba −13* + 16-) and two of the *Glu-D1* locus (*Glu-D1d −5 + 10-* and *Glu-D1ap −2.5 + 12-*) did not appeared among the accessions collected in 2004 [36]. Consequently, the retrieval of the accession from 1930s collection that contained the allele *Glu-D1an* (PI 348672) is clearly necessary as line per se or as donor of this interesting allele. Although limited for the low number of spelt lines used, the studies carried out by Rodriguez-Quijano et al. [96] and Callejo et al. [97] confirmed the good relationship of this allele with baking quality in spelt.

This variation was also higher for other seed storage proteins, detecting up to 60 patterns for the low-molecular-weight glutenin subunits (LMWGs) [86], 61 for ω-/γ-gliadins (*Gli-1* loci) and 19 for β-/α-gliadins (*Gli-2* loci) [88]. In these cases, notable differences in the frequency of each pattern were detected, being considerate some of these patterns as rare (frequency ≤ 5%) or as very rare (frequency ≤ 1%). This could be related with the usual technique used to manipulate these materials. All processes carried out with these old materials are mainly performed by hand, and so some combinations may have been missed when the materials were mechanically manipulated.

Among the starch synthases in wheat, the granule-bound starch synthase or waxy protein has been the most studied. This enzyme is the only one involved in amylose synthesis, and its presence/absence, together with its functionality, determines the final starch composition and properties [81]. Consequently, this enzyme has been also studied in spelt with the main aim to detect the presence of spelt waxy type (≈0% amylose).

The first study on this protein in Spanish spelt wheat was by Rodriguez-Quijano et al. [98], who evaluated 144 accessions from Spain, detecting variation in two of the three genes: two variants for *Wx-A1* and three for *Wx-B1*. Then, another spelt collection of 420 Spanish accessions was studied using waxy protein separation in SDS-PAGE, determining variation for the three *Wx* genes [99]. However, some of these alleles could be classified as rare or very rare and are at clear risk of disappearing due to stochastic processes of genetic drift.

However, due to the size of these proteins, their study can be difficult and many internal variations can be masked. This may be partially resolved by evaluating the nucleotide sequences of the synthesized genes. The PCR amplification of these genes in three overlapping fragments from genomic DNA has notably increased the variation detected for these genes [81]. This variation is a consequence of the fragmentary structure (11 introns and 12 exons) of the *Wx* genes [100], with most of the changes occurring in the introns. However, this does not influence protein size, and amino acid changes within exons generally have low impact on the functionality of these enzymes [81].

Some of these new alleles were detected in spelt [101], including two clear types for the *Wx-B1* gene: types I and II. Type I could be classified as the wild type and includes numerous variants similar to those found in common wheat (*Wx-A1a*, *Wx-B1a* and *Wx-D1a*). In contrast, type II presents some clear differences to type I with 14 amino acid changes, nine of them inside the mature protein [101]. Molecular analysis of these alleles showed that this change could be related to the synthesis of tetraploid wheat (≈0.6 million years ago). This suggested the presence of two types within the Spanish spelt pool, which is consistent with the old hypothesis on the two origins in the Iberian group [10]. This *Wx-B1* type II gene is not exclusive to spelt and has also been detected in old landraces of Spanish common wheat [102]. This suggests that these common wheat landraces derived from these spelt lines due to domestication events.

Within these materials, one allelic variant with low functionality due to presence of a transposon-like sequence inside the 5th intron was also detected, which generates an aberrant splicing [103]. This type of allele opens the possibility of developing intermediate materials among the waxy (≈0% amylose) and the partial waxy types (≈20% amylose). For this reason, we coined the term “quasi-waxy” for these potential materials [103].

In contrast, puroindoline variation in spelt has been little studied, probably because these materials generally show a soft texture. No spelt accessions carrying alleles differing from the wild alleles (*Pina-D1a* and *Pinb-D1a*) have been reported at now.

## 6. Recovery or Development of New Spelt Varieties

Parallel with the return to more natural food, the interest in traditional products has clearly increased. However, in many cases, these products are currently made with modern flours, and so some properties associated with these traditional products differ. For this reason, the renewed interest in these products has increased the need to recover the old materials used to make them in the past, together with investigation of the quality characteristics of these old materials to design new materials with similar characteristics.

Faced with these circumstances, two ways have been used with more or less success: use of old landraces vs. generating new varieties by crosses with common wheat. The main way is evaluating old landraces and identifying the best lines. This process implies that phenotypic selection within landraces, together with correction of undesirable traits (mainly the lodging due to the high plant height), has been carried out by cultural techniques: low fertilization or increasing seeding density. Spelt has been grown in some European regions with traditions in the maintenance of spelt until recent times. Some of these zones have designed protection mechanisms for these ancestral products regulated by European Union laws, such as the Protected Geographical Indication (PGI), which is key to the maintenance of pure spelt. One example of this is the PGI «*Farro della Garfagnana*» (Italy) [104]. One similar process has been valued in several occasions for the Asturian spelt (*escanda asturiana*), although without positive results until moment.

**Figure 6:**
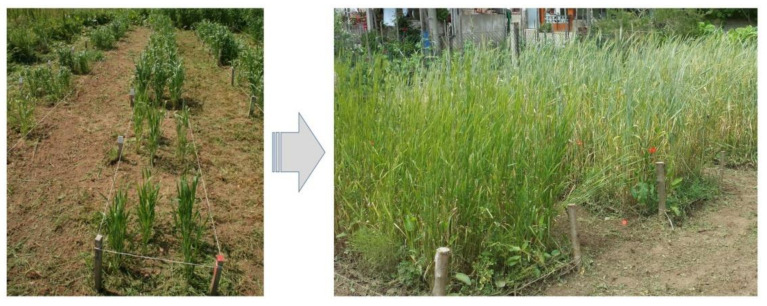
Recuperation of old landraces in Asturias (Northern Spain).

Our team developed one set of 32 lines using this way, which were obtained from different Spanish landraces included in the 1930s collection, and selected according with the HMWGs composition [85]. For this, one unique seed was used to generate each line, removing any possible heterogeneity present in the landraces used (Figure 6). The additional selection among these materials has allowed the identification of one promising line with good adaptation to the climatic and edaphic conditions of Asturias (unpublished results). This tendency to local adaptation is frequent in traditional materials, and makes selection difficult in other environments.

**Figure 7:**
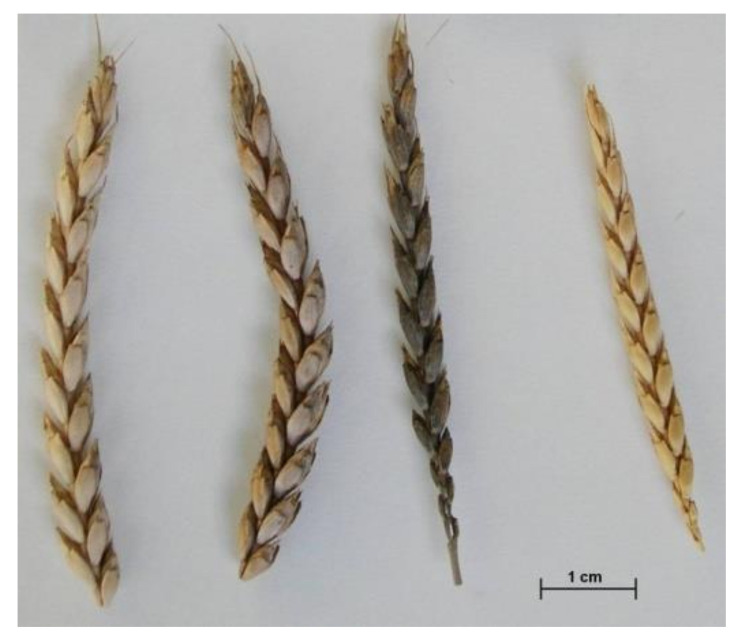
Spikes of spelt × common wheat hybrids found in Asturias during the collecting mission of 2004.

The second way to develop spelt varieties is generating hybrids by crosses with common wheat (Figure 7) [105,106]. For the development of lines from these hybrids, the offsprings should be self-pollinated during several genenerations, selecting those plants that, additionally to good agronomical traits, present spikes with speltoid morphology (allele *q*) and tenacious glumes (allele *Tg*) (Figure 1). This process, in general long in the time, can be shorted using speed breeding techniques [107]. Alternatively, the anther culture for doubled haploid production can be used with this same finality [108,109,110].

The main objective of the new spelt varieties is the correction of some agronomical problems such as lodging by producing shorter plants, which permit an increase in harvest index [111]. These materials have been obtained by hybridization with semi-dwarf common wheat, and European cultivars as Alkor, Badengold, Ceralio and Cosmos are good examples. In any case, the resulting varieties should maintain morphologic characteristics similar to traditional spelt, including spike morphology and tenacious glumes, because without these specific characteristics they cannot be considered to be spelt. In any case, the studies carried out with these new spelt varieties showed that, although their grain yields are clearly higher than those traditional spelt, these are sensitive lower than those of common wheat [61,62,112].

There has so far been little development of new spelt varieties (deriving from crosses with common wheat) in Spain or with Spanish germplasm, with two cultivars being registered: Annamaria and Viso. However, this could be important in coming years due to the renewed interest in this crop. In fact, new materials are being developed using both the double-haploid production [113] and the self-pollination and selection by speed breeding (unpublished results). Nevertheless, the likelihood of genetic erosion in the traditional material due to the presence of these new materials should be considered. Obviously, agriculture is an economic activity, and the loss of profitability could motivate some traditional growers to neglect the old material for more modern spelt that, a priori, could be more profitable due to lower crop losses. In this case, traditional spelt would only be cultivated by the few aged farmers who continue to use it for home consumption and could be permanently lost when they pass.

## 7. An Alternative Crop vs. a Miracle Crop

The best conservation method is use and, consequently, the renewed interest in ancient crops can generate maintenance of these genetic resources. However, this process has gone hand-in-hand with numerous myths about the benefits of these old crops, which could have the opposite effect. Paradoxically, some of these ancient crops were not considered desirable in antiquity. For example, Dioscorides in his “*De Materia Medica*” (On Medical Materia) wrote on spelt that “*…* *made into bread it is less nourishing than wheat*” [114]. Obviously, the current canon differs from that of those previous times, but this should help us to understand that there are no miracle crops.

According to the popular press, social media and biased scientific studies, ancient wheats differ from modern wheat in their composition of bioactive components related to nutrition and health. Nevertheless, the scientific data indicate that ancient wheats differ little from modern wheat species in the contents of several bioactive components, including phenolic compounds, vitamins and dietary fiber, with this last component generally at lower levels in ancient than in modern wheats [115]. In any case, because few genotypes have been analyzed for grain components related to nutrition and health, and represent probably less than 1% of the genetic resources available [115,116], all this information should be evaluated with caution.

Another false belief is that spelt is a non-cytotoxic material that can be consumed without precaution by persons with immunoreactions to gluten or other wheat components. Obviously, this is not true and, although some studies have been carried out [116,117,118,119], the data obtained are scarce and conclusions are at best preliminary. Furthermore, most of the consumed spelt is derived from crosses with common wheat, considered highly dangerous for those with celiac disease; furthermore, some data suggest that traditional spelt is no less toxic than common wheat [120,121]. For other human diseases, such as irritable bowel syndrome or Type 2 diabetes, the positive effect could be more related to the processing conditions of these ancient wheats, such as long periods of fermentation or the absence of additives [122,123].

The main utility of these ancient wheats is the making of traditional foods. In this regard, it is also very important to correctly classify these products, clearly differentiating those that are making only with spelt from those that contained only one part of spelt. The consumer must be able to adequately assess the virtues of the product, and this is difficult if in addition to products made with traditional or modern spelt, we also have products that contain only spelt or variable amounts of spelt flour.

In recent times, a campaign against wheat has developed in various sectors [124], ignoring 5000 years of history and the proven virtues associated with its consumption [125], and movements have emerged that intend to turn ancestral wheats into alternatives to common wheat. This is misguided because these ancient wheats are neither better nor worse than modern wheat, but complementary to the available cereals. It is essential not to push these species to a second and definitive extinction, that spelt becomes one more cereal, without the intention of mythologizing it or promoting it as an impossible substitute for wheat.

## 8. Final Remarks

In recent times, spelt has become a fashionable crop in some areas of Europe, including Spain. Although this species was widely cultivated in the past in Spain, during the 20th century it suffered deep erosion that led it to near extinction [32]. This deep erosion caused that the available material was mostly stored in some germplasm banks, being scarce in the fields [35].

The revival of spelt cultivation at recent decades did mainly stock up from the scarce material available by farmers, which implied a very narrow genetic base. For this reason, it is necessary to evaluate and recover materials from germplasm banks, which have not been accessible for pioneer farmers in the recovery of spelt as a crop.

Nowadays, similar to what has been performed in other parts of Europe [56,126,127,128,129,130]; spelt improvement programs have been developed in Spain. Although some of them have been carried out by the selection within traditional materials [97,98], others have used the hybridization with semi-dwarf common wheat in order to obtain spelt varieties with characteristics agronomic more suitable to current agricultural techniques [113]. These new varieties present low-height plants that allow their mechanization and avoid the habitual lodging in traditional materials. Nevertheless, regardless of the fact that the growing consumer demand has led to a gradual increase in the cultivated area, it still remains at low levels, so there are no official statistics in this regard.

This trend must be linked to conservation programs of the genetic resources of the species, to avoid possible erosion and to develop higher variation levels in the commercialized materials. Furthermore, the studies carried out to assess the variability levels of the materials deposited in germplasm banks have shown not only the uniqueness of the material from the Iberian Peninsula [10], but also the presence of some variation levels for, among others, technological quality characteristics [37,84,85,86,87,88,94,98,99,101,103], which may allow an important diversity within the varieties of spelt available.

Due to the current trend to seek additional nutritional characteristics in crops, the development of spelt varieties with nutraceutical characteristics due to the presence of trace elements, can be a good tool for the maintenance and expansion of this crop both in Spain and elsewhere of the world.

## Figures and Tables

**Table 1:** Botanical varieties found into the spelt collection according to Lagasca and Clemente [33].

Group	Glumes Types	Glumes Colour	Botanical Variety
*mutica*	Glabrous	White	var. *album* (Alef.) Körn.
	Glabrous	Red	var. *duhamelianum* (Mazz.) Koern.
*aristata*	Glabrous	White	var. *arduinii* (Mazz.) Körn.
	Glabrous	Red	var. *vulpinum* (Alef.) Körn.
	Pubescent	White	var. *albivelutinum* Körn.
	Pubescent	Red	var. *rubrivelutinum* Körn.
	Pubescent	Black or Blue	var. *caeruleum* (Alef.) Körn.

## Data Availability

Not applicable.

## References

[1] Zohary D., Hopf M. (2000). Domestication of Plants in the Old World: The Origin and Spread of Cultivated Plants in West Asia, Europe, and the Nile Valley.

[2] Harlan J.R. (1992). Crops and Man.

[3] Giles R.J., Brown T.A. (2006). GluDy allele variations in Aegilops tauschii and Triticum aestivum: Implications for the origins of hexaploid wheats. Theor. Appl. Genet..

[4] McFadden E.S., Sears E.R. (1945). The artificial synthesis of Triticum spelta. Genetics.

[5] Salamini F., Ozkan H., Brandolini A., Schafer-Pregl R., Martin W. (2002). Genetics and geography of wild cereal domestication in the near east. Nat. Rev. Genet..

[6] Liu Y.G., Tsunewaki K. (1991). Restriction-fragment-length-polymorphism (RFLP) analysis in wheat. 2. Linkage maps of the RFLP sites in common wheat. Jpn. J. Genet..

[7] Yan Y., Hsam S.L.K., Yu J.Z., Jiang Y., Ohtsuka I., Zeller F.J. (2003). HMW and LMW glutenin alleles among putative tetraploid and hexaploid European spelt wheat (Triticum spelta L.) progenitors. Theor. Appl. Genet..

[8] Blatter R.H.E., Jacomet S., Schlumbaum A. (2004). About the origin of European spelt (Triticum spelta L.): Allelic differentiation of the HMW Glutenin B1-1 and A1-2 subunit genes. Theor. Appl. Genet..

[9] Dedkova O.S., Badaeva E.D., Mitrofanova O.P., Zelenin A.V., Pukhalskiy V.A. (2004). Analysis of intraspecific divergence of hexaploid wheat Triticum spelta L. by C-banding of chromosomes. Russ. J. Genet..

[10] Elía M., Moralejo M., Rodriguez-Quijano M., Molina-Cano J.L. (2004). Spanish spelt: A separate gene pool within the spelt germplasm. Plant Breed..

[11] Muramatsu M. (1985). Spike type in two cultivars of Triticum dicoccum with the spelta gene q compared with the Q-bearing variety liguliforme. Jpn. J. Breed..

[12] Kato K., Miura H., Sawada S. (1999). QTL mapping of genes controlling ear emergence time and plant height on chromosome 5A of wheat. Theor. Appl. Genet..

[13] Kato K., Sonokawa R., Miura H., Sawada S. (2003). Dwarfing effect associated with the threshability gene Q on wheat chromosome 5A. Plant Breed..

[14] Simons K.J., Fellers J.P., Trick H.N., Zhang Z., Tai Y.-S., Gill B.S., Faris J.D. (2006). Molecular characterization of the major wheat domestication gene Q. Genetics.

[15] Zhang Z.C., Belcram H., Gornicki P., Charles M., Just J., Huneau C., Magdelenat G., Couloux A., Samain S., Gill B.S. (2011). Duplication and partitioning in evolution and function of homoeologous Q loci governing domestication characters in polyploid wheat. Proc. Natl. Acad. Sci. USA.

[16] Debernardi J.M., Lin H., Chuck G., Faris J.D., Dubcovsky J. (2017). microRNA172 plays a crucial role in wheat spike morphogenesis and grain threshability. Development.

[17] Greenwood J.R., Finnegan E.J., Watanabe N., Trevaskis B., Swain S.M. (2017). New alleles of the wheat domestication gene Q reveal multiple roles in growth and reproductive development. Development.

[18] Kato K., Miura H., Akiyama M., Kuroshima M., Sawada S. (1998). RFLP mapping of the three major genes, Vrn1, Q and B1, on the long arm of chromosome 5A of wheat. Euphytica.

[19] Kerber E.R., Rowland G.G. (1974). Origin of the free threshing character in hexaploid wheat. Can. J. Genet. Cytol..

[20] Simonetti M.C., Bellomo M.P., Laghetti G., Perrino P., Simeone R., Blanco A. (1999). Quantitative trait loci influencing free-threshing habit in tetraploid wheats. Genet. Resour. Crop Evol..

[21] Dvorak J., Deal K.R., Luo M.C., You F.M., von Borstel K., Dehghani H. (2012). The origin of spelt and free-threshing hexaploid wheat. J. Hered..

[22] Faris J., Zhang Q., Chao S., Zhang Z., Xu S. (2014). Analysis of agronomic and domestication traits in a durum × cultivated emmer wheat population using a high-density single nucleotide polymorphism-based linkage map. Theor. Appl. Genet..

[23] Campbell K.G. (1997). Spelt: Agronomy, genetics, and breeding. Plant Breed. Rev..

[24] Jacomet S. (2006). Identification of Cereal Remains from Archaeological Sites.

[25] Brown T.A., Allaby R.G., Brown K.A., Odonoghue K., Sallares R. (1994). DNA in wheat seeds from European archaeological sites. Experientia.

[26] Brown T.A., Cappellini E., Kistler L., Lister D.L., Oliveira H.R., Wales N., Schlumbaum A. (2015). Recent advances in ancient DNA research and their implications for archaeobotany. Veget. Hist. Archaeobot..

[27] Maron L. (2019). Sequencing of ancient wheat genomes opens a window into the past. Plant J..

[28] Harlan J.R., Evans L.T., Peacock W.J. (1981). The early history of wheat: Earliest traces to the sack of Rome. Wheat Science-Today and Tomorrow.

[29] Nesbitt M., Samuel D., Padulosi S., Hammer K., Heller J. (1996). From staple crop to extinction? The archaeology and history of hulled wheat. Hulled Wheats.

[30] Buxó i Capdevila R., Alonso N., Canal D., Echave C., Gonzalez I. (1997). Archaeobotanical remains of hulled and naked cereals in the Iberian Peninsula. Veget. Hist. Archaeobot..

[31] Peña-Chocarro L., Pérez- Jordà G., Alonso N., Antolín F., Teira-Brión A., Tereso J.P., Montes Moya E.M., López Reyes D. (2019). Roman and medieval crops in the Iberian Peninsula: A first overview of seeds and fruits from archaeological sites. Quat. Int..

[32] Peña-Chocarro L., Padulosi S., Hammer K., Heller J. (1996). In situ conservation of hulled wheat species: The case of Spain. Hulled Wheats.

[33] Tellez-Molina R., Alonso-Peña M. (1952). Los Trigos de la Ceres Hispanica de Lagasca y Clemente.

[34] Alvargonzalez C. (1908). La Escanda, su Origen y su Cultivo.

[35] Caballero L., Martín L.M., Alvarez J.B. (2007). Agrobiodiversity of hulled wheats in Asturias (North of Spain). Genet. Resour. Crop Evol..

[36] Caballero L., Martín L.M., Alvarez J.B. (2008). Genetic diversity in Spanish populations of Triticum spelta L. (escanda): Example of an endangered genetic resource. Genet. Resour. Crop Evol..

[37] Alvarez J.B., Caballero L., Martín L.M. (2007). Variability for morphological traits and high molecular weight glutenin subunits in Spanish spelt lines. Plant Genet. Resour.-Charact. Util..

[38] McFadden E.S., Sears E.R. (1946). The origin of Triticum spelta and its free-threshing hexaploid relatives. J. Hered..

[39] McFadden E.S., Sears E.R. (1946). The origin of Triticum spelta and its free-threshing hexaploid relatives. J. Hered..

[40] Zeven A.C., Turkenst L.J., Stubbs R.W. (1968). Spelt (Triticum spelta L.) as a possible source of race-non-specific resistance to yellow rust (Puccinia striiformis West.). Euphytica.

[41] Zeller F.J., Felsenstein F.G., Lutz J., Holzerland A., Katzhammer M., Kellermann A., Kunzler J., Stephan U., Oppitz K. (1994). Studies on the resistance of several spelt wheat (*Triticum aestivum* (L.) Thell. ssp. spelta (L.) Thell.) cultivars against wheat powdery mildew (*Erysiphe graminis* f. sp. tritici) and leaf rust of wheat (Puccinia recondita f. sp. tritici). Bodenkultur.

[42] Dyck P.L., Sykes E.E. (1994). Genetics of leaf-rust resistance in three spelt wheats. Can. J. Plant Sci..

[43] Singh D., Mohler V., Park R.F. (2013). Discovery, characterisation and mapping of wheat leaf rust resistance gene Lr71. Euphytica.

[44] Peng F., Song N., Shen H., Wu H., Dong H., Zhang J., Li Y., Peng H., Ni Z., Liu Z. (2014). Molecular mapping of a recessive powdery mildew resistance gene in spelt wheat cultivar Hubel. Mol. Breed..

[45] Mohler V., Singh D., Singrun C., Park R.F. (2012). Characterization and mapping of Lr65 in spelt wheat ‘Altgold Rotkorn’. Plant Breed..

[46] Dinkar V., Jha S.K., Mallick N., Niranjana M., Agarwal P., Sharma J.B. (2020). Vinod, Molecular mapping of a new recessive wheat leaf rust resistance gene originating from Triticum spelta. Sci. Rep..

[47] McVey D.V., Leonard K.J. (1990). Resistance to wheat stem rust in spring spelts. Plant Dis..

[48] Goriewa-Duba K., Duba A., Suchowilska E., Wiwart M. (2020). An analysis of the genetic diversity of bread wheat × spelt breeding lines in terms of their resistance to powdery mildew and leaf rust. Agronomy.

[49] Singh P.K., Mergoum M., Ali S., Adhikari T.B., Elias E.M., Hughes G.R. (2006). Identification of new sources of resistance to tan spot, Stagonospora nodorum blotch, and Septoria tritici blotch of wheat. Crop Sci..

[50] Simon M.R., Khlestkina E.K., Castillo N.S., Borner A. (2010). Mapping quantitative resistance to septoria tritici blotch in spelt wheat. Eur. J. Plant Pathol..

[51] Trichopoulou A., Lagiou P. (1997). Healthy traditional Mediterranean diet: An expression of culture, history, and lifestyle. Nutr. Rev..

[52] Arzani A., Ashraf M. (2017). Cultivated ancient wheats (Triticum spp.): A potential source of health-beneficial food products. Compr. Rev. Food Sci. Food Saf..

[53] Lăcătușu C.-M., Grigorescu E.-D., Floria M., Onofriescu A., Mihai B.-M. (2019). The Mediterranean diet: From an environment-driven food culture to an emerging medical prescription. Int. J. Environ. Res. Public Health.

[54] Singh A., Banerjee P., Anas M., Singh N., Qamar I. (2020). Traditional nutritional and health practices targeting lifestyle behavioral changes in humans. J. Lifestyle Med..

[55] Geisslitz S., Scherf K.A. (2020). Rediscovering ancient wheats. Cereal Foods World.

[56] Longin C.F.H., Würschum T. (2014). Genetic variability, heritability and correlation among agronomic and disease resistance traits in a diversity panel and elite breeding material of spelt wheat. Plant Breed..

[57] Konvalina P., Capouchová I., Stehno Z., Moudrý J. (2010). Agronomic characteristics of the spring forms of the wheat landraces (einkorn, emmer, spelt, intermediate bread wheat) grown in organic farming. J. Agrobiol..

[58] Krystyna Ż.-G., Katarzyna M., Józef T., Janusz G. (2018). Physical and technological properties of kernels and flour made from spelt grown in an organic farming system in north-eastern Poland. J. Cereal Sci..

[59] Ugrenovic V., Bodroza-Solarov M., Pezo L., Djisalov J., Popovic V., Maric B., Filipovic V. (2018). Analysis of spelt variability (Triticum spelta L.) grown in different conditions of Serbia by organic conditions. Genetika.

[60] Engert N., Honermeier B. (2012). Characterization of grain quality and phenolic acids in ancient wheat species (Triticum sp.). J. Appl. Bot. Food Qual..

[61] Rachoń L., Bobryk-Mamczarz A., Kiełtyka-Dadasiewicz A. (2020). Hulled wheat productivity and quality in modern agriculture against conventional wheat species. Agriculture.

[62] Biel W., Jaroszewska A., Stankowski S., Sobolewska M., Kępińska-Pacelik J. (2021). Comparison of yield, chemical composition and farinograph properties of common and ancient wheat grains. Eur. Food Res. Technol..

[63] Kulathunga J., Reuhs B.L., Zwinger S., Simsek S. (2021). Comparative study on kernel quality and chemical composition of ancient and modern wheat species: Einkorn, emmer, spelt and hard red spring wheat. Foods.

[64] Kulathunga J., Reuhs B.L., Simsek S. (2020). A review: Novel trends in hulled wheat processing for value addition. Trends Food Sci. Technol..

[65] Cubadda R., Marconi E., Belton P.S., Taylor J.R.N. (2002). Spelt wheat. Pseudocereals and Less Common Cereals: Grain Properties and Utilization Potential.

[66] Cubadda R., Marconi E., Padulosi S., Hammer K., Heller J. (1996). Technological and nutritional aspects in emmer and spelt. Hulled Wheats.

[67] White P.J., Broadley M.R. (2005). Biofortifying crops with essential mineral elements. Trends Plant Sci..

[68] Bouis H.E., Hotz C., McClafferty B., Meenakshi J.V., Pfeiffer W.H. (2011). Biofortification: A new tool to reduce micronutrient malnutrition. Food Nutr. Bull..

[69] Abdel-Aal E.S.M., Hucl P., Sosulski F.W. (1995). Compositional and nutritional characteristics of spring einkorn and spelt wheats. Cereal Chem..

[70] Piergiovanni A.R., Rizzi R., Pannacciulli E., Della Gatta C. (1997). Mineral composition in hulled wheat grains: A comparison between emmer (Triticum dicoccon Schrank) and spelt (T. spelta L.) accessions. Int. J. Food Sci. Nutr..

[71] Grela E.R. (1996). Nutrient composition and content of antinutritional factors in spelt (Triticum spelta L.) cultivars. J. Sci. Food Agric..

[72] Ranhotra G.S., Gelroth J.A., Glaser B.K., Lorenz K.J. (1995). Baking and nutritional qualities of a spelt wheat sample. Lebensm. Wiss. Technol..

[73] Ruibal-Mendieta N.L., Delacroix D.L., Mignolet E., Pycke J.M., Marques C., Rozenberg R., Petitjean G., Habib-Jiwan J.L., Meurens M., Quetin-Leclercq J. (2005). Spelt (Triticum aestivum ssp. spelta) as a source of breadmaking flours and bran naturally enriched in oleic acid and minerals but not phytic acid. J. Agric. Food Chem..

[74] Gomez-Becerra H.F., Erdem H., Yazici A., Tutus Y., Torun B., Ozturk L., Cakmak I. (2010). Grain concentrations of protein and mineral nutrients in a large collection of spelt wheat grown under different environments. J. Cereal Sci..

[75] Srinivasa J., Arun B., Mishra V.K., Chand R., Sharma D., Bhardwaj S.C., Joshi A.K. (2014). Accessing spelt gene pool to develop well-adapted zinc- and iron-rich bread wheat. Crop Sci..

[76] Srinivasa J., Arun B., Mishra V., Singh G., Velu G., Babu R., Vasistha N., Joshi A. (2014). Zinc and iron concentration QTL mapped in a Triticum spelta × T. aestivum cross. Theor. Appl. Genet..

[77] Guzmán C., Medina-Larqué A.S., Velu G., González-Santoyo H., Singh R.P., Huerta-Espino J., Ortiz-Monasterio I., Peña R.J. (2014). Use of wheat genetic resources to develop biofortified wheat with enhanced grain zinc and iron concentrations and desirable processing quality. J. Cereal Sci..

[78] Rewers M. (2005). Epidemiology of celiac disease: What are the prevalence, incidence, and progression of celiac disease?. Gastroenterology.

[79] Sapone A., Lammers K.M., Casolaro V., Cammarota M., Giuliano M.T., De Rosa M., Stefanile R., Mazzarella G., Tolone C., Russo M.I. (2011). Divergence of gut permeability and mucosal immune gene expression in two gluten-associated conditions: Celiac disease and gluten sensitivity. BMC Med..

[80] Ozuna C.V., Iehisa J.C.M., Giménez M.J., Alvarez J.B., Sousa C., Barro F. (2015). Diversification of the celiac disease α-gliadin complex in wheat: A 33-mer peptide with six overlapping epitopes, evolved following polyploidization. Plant J..

[81] Guzmán C., Alvarez J.B. (2016). Wheat waxy proteins: Polymorphism, molecular characterization and effects on starch properties. Theor. Appl. Genet..

[82] Wrigley C., Békés F., Bushuk W. (2006). Gliadin and Glutenin: The Unique Balance of Wheat Quality.

[83] Morris C.F. (2002). Puroindolines: The molecular genetic basis of wheat grain hardness. Plant Mol. Biol..

[84] Rodriguez-Quijano M., Vazquez J.F., Carrillo J.M. (1990). Variation of high molecular weight glutenin subunits in Spanish landraces of Triticum aestivum ssp. vulgare and ssp. spelta. J. Genet. Breed..

[85] Caballero L., Martín L.M., Alvarez J.B. (2001). Allelic variation of the HMW glutenin subunits in Spanish accessions of spelt wheat (Triticum aestivum ssp spelta L. em. Thell.). Theor. Appl. Genet..

[86] Caballero L., Martín L.M., Alvarez J.B. (2004). Genetic variability of the low-molecular-weight glutenin subunits in spelt wheat (Triticum aestivum ssp spelta L. em Thell.). Theor. Appl. Genet..

[87] Caballero L., Martín L.M., Alvarez J.B. (2004). Intra- and interpopulation diversity for HMW glutenin subunits in Spanish spelt wheat. Genet. Resour. Crop Evol..

[88] Caballero L., Martín L.M., Alvarez J.B. (2004). Variation and genetic diversity for gliadins in Spanish spelt wheat accessions. Genet. Resour. Crop Evol..

[89] Payne P.I., Holt L.M., Jackson E.A., Law C.N. (1984). Wheat storage proteins: Their genetics and their potential for manipulation by plant breeding. Phil. Trans. R. Soc. Lond. Ser. B.

[90] Payne P.I., Lawrence G.J. (1983). Catalogue of alleles for the complex gene loci, Glu-A1, Glu-B1 and Glu-D1 which code for high-molecular-weight subunits of glutenin in hexaploid wheat. Cereal Res. Commun..

[91] Lawrence G.J. (1986). The high-molecular-weight glutenin subunit composition of Australian wheat cultivars. Aust. J. Agric. Res..

[92] Lukow O.M., Payne P.I., Tkachuk R. (1989). The HMW glutenin subunit composition of Canadian wheat cultivars and their association with bread-making quality. J. Sci. Food Agric..

[93] Ayala M., Guzmán C., Peña R.J., Alvarez J.B. (2016). Diversity of phenotypic (plant and grain morphological) and genotypic (glutenin alleles in Glu-1 and Glu-3 loci) traits of wheat landraces (Triticum aestivum) from Andalusia (Southern Spain). Genet. Resour. Crop Evol..

[94] Caballero L., Martín L.M., Alvarez J.B. (2008). Relationships between the HMW- and LMW-glutenin subunits and SDS-sedimentation volume in spanish hulled wheat lines. Czech J. Genet. Plant Breed..

[95] Dick J.W., Quick J.S. (1983). A modified screening test for rapid estimation of gluten strength in early-generation durum wheat breeding lines. Cereal Chem..

[96] Rodríguez-Quijano M., Vargas-Kostiuk M.-E., Ribeiro M., Callejo M.J. (2019). *Triticum aestivum* ssp. vulgare and ssp. spelta cultivars. 1. Functional evaluation. Eur. Food Res. Technol..

[97] Callejo M.J., Vargas-Kostiuk M.-E., Ribeiro M., Rodríguez-Quijano M. (2019). *Triticum aestivum* ssp. vulgare and ssp. spelta cultivars: 2. Bread-making optimisation. Eur. Food Res. Technol..

[98] Rodriguez-Quijano M., Nieto-Taladriz M.T., Carrillo J.M. (1998). Polymorphism of waxy proteins in Iberian hexaploid wheats. Plant Breed..

[99] Guzmán C., Caballero L., Moral A., Alvarez J.B. (2010). Genetic variation for waxy proteins and amylose content in Spanish spelt wheat (Triticum spelta L.). Genet. Resour. Crop Evol..

[100] Yan L., Bhave M., Fairclough R., Konik C., Rahman S., Appels R. (2000). The genes encoding granule-bound starch synthases at the waxy loci of the A, B, and D progenitors of common wheat. Genome.

[101] Guzmán C., Caballero L., Martín L.M., Alvarez J.B. (2012). Waxy genes from spelt wheat: New alleles for modern wheat breeding and new phylogenetic inferences about the origin of this species. Ann. Bot..

[102] Alvarez J.B., Castellano L., Huertas-García A.B., Guzmán C. (2021). Molecular characterization of five novel Wx-A1 alleles in common wheat including one silent allele by transposon insertion. Plant Sci..

[103] Guzmán C., Caballero L., Yamamori M., Alvarez J.B. (2012). Molecular characterization of a new waxy allele with partial expression in spelt wheat. Planta.

[104] Fischler F. (1997). Commission Regulation (EC) No 1263/96 of 1 July 1996 supplementing the Annex to Regulation (EC) No 1107/96 on the registration of geographical indications and designations of origin under the procedure laid down in Article 17 of Regulation (EEC) No 2081/92. Off. J. Eur. Communities.

[105] Schmid J., Winzeler H. (1990). Genetic studies of crosses between common wheat (Triticum aestivum L.) and spelt (Triticum spelta L.). J. Genet. Breed..

[106] Winzeler H., Schmid J.E., Winzeler M. (1994). Analysis of the yield potential and yield components of F_1_ and F_2_ hybrids of crosses between wheat (Triticum aestivum L.) and spelt (Triticum spelta L.). Euphytica.

[107] Watson A., Ghosh S., Williams M.J., Cuddy W.S., Simmonds J., Rey M.D., Asyraf Md Hatta M., Hinchliffe A., Steed A., Reynolds D. (2018). Speed breeding is a powerful tool to accelerate crop research and breeding. Nat. Plants.

[108] Lantos C., Bóna L., Nagy É., Békés F., Pauk J. (2018). Induction of in vitro androgenesis in anther and isolated microspore culture of different spelt wheat (Triticum spelta L.) genotypes. Plant Cell Tissue Organ Cult..

[109] Lantos C., Purgel S., Ács K., Langó B., Bóna L., Boda K., Békés F., Pauk J. (2019). Utilization of in vitro anther culture in spelt wheat breeding. Plants.

[110] Lantos C., Pauk J., Segui-Simarro J.M. (2021). In vitro anther culture for doubled haploid plant production in spelt wheat. Doubled Haploid Technology: Volume 1: General Topics, Alliaceae, Cereals.

[111] Keller M., Karutz C., Schmid J.E., Stamp P., Winzeler M., Keller B., Messmer M.M. (1999). Quantitative trait loci for lodging resistance in a segregating wheat × spelt population. Theor. Appl. Genet..

[112] Longin C.F.H., Ziegler J., Schweiggert R., Koehler P., Carle R., Würschum T. (2016). Comparative study of hulled (einkorn, emmer, and spelt) and naked wheats (durum and bread wheat): Agronomic performance and quality traits. Crop Sci..

[113] Castillo A.M., Allue S., Costar A., Alvaro F., Valles M.P. (2019). Doubled haploid production from Spanish and Central European spelt by anther culture. J. Agr. Sci. Technol..

[114] Dioscorides P., Valdés M.G. (1998). De Materia Medica (Plantas y Remedios Medicinales).

[115] Shewry P.R., Hey S. (2015). Do “ancient” wheat species differ from modern bread wheat in their contents of bioactive components?. J. Cereal Sci..

[116] Dinu M., Whittaker A., Pagliai G., Benedettelli S., Sofi F. (2018). Ancient wheat species and human health: Biochemical and clinical implications. J. Nutr. Biochem..

[117] Shewry P.R. (2018). Do ancient types of wheat have health benefits compared with modern bread wheat?. J. Cereal Sci..

[118] Vincentini O., Maialetti F., Gazza L., Silano M., Dessi M., De Vincenzi M., Pogna N.E. (2007). Environmental factors of celiac disease: Cytotoxicity of hulled wheat species Triticum monococcum, *T. turgidum* ssp. dicoccum and *T. aestivum* ssp. spelta. J. Gastroenterol. Hepatol..

[119] Šuligoj T., Gregorini A., Colomba M., Ellis H.J., Ciclitira P.J. (2013). Evaluation of the safety of ancient strains of wheat in coeliac disease reveals heterogeneous small intestinal T cell responses suggestive of coeliac toxicity. Clin. Nutr..

[120] Zanini B., Petroboni B., Not T., Di Toro N., Villanacci V., Lanzarotto F., Pogna N., Ricci C., Lanzini A. (2013). Search for atoxic cereals: A single blind, cross-over study on the safety of a single dose of Triticum monococcum, in patients with celiac disease. BMC Gastroenterol..

[121] Van den Broeck H.C., de Jong H.C., Salentijn E.M.J., Dekking L., Bosch D., Hamer R.J., Gilissen L.J.W.J., van der Meer I.M., Smulders M.J.M. (2010). Presence of celiac disease epitopes in modern and old hexaploid wheat varieties: Wheat breeding may have contributed to increased prevalence of celiac disease. Theor. Appl. Genet..

[122] Kucek L.K., Veenstra L.D., Amnuaycheewa P., Sorrells M.E. (2015). A grounded guide to gluten: How modern genotypes and processing impact wheat sensitivity. Compr. Rev. Food Sci. Food Saf..

[123] Guerrini L., Parenti O., Angeloni G., Zanoni B. (2019). The bread making process of ancient wheat: A semi-structured interview to bakers. J. Cereal Sci..

[124] Jones J.M. (2012). Wheat belly—An analysis of selected statements and basic theses from the book. Cereal Foods World.

[125] Shewry P.R., Charmet G., Branlard G., Lafiandra D., Gergely S., Salgó A., Saulnier L., Bedő Z., Mills E.N.C., Ward J.L. (2012). Developing new types of wheat with enhanced health benefits. Trends Food Sci. Technol..

[126] Rapp M., Beck H., Gütler H., Heilig W., Starck N., Römer P., Cuendet C., Uhlig F., Kurz H., Würschum T. (2017). Spelt: Agronomy, quality, and flavor of its breads from 30 varieties tested across multiple environments. Crop Sci..

[127] Packa D., Załuski D., Graban Ł., Lajszner W. (2019). An evaluation of spelt crosses for breeding new varieties of spring spelt. Agronomy.

[128] Ratajczak K., Sulewska H., Grażyna S., Matysik P. (2020). Agronomic traits and grain quality of selected spelt wheat varieties versus common wheat. J. Crop Improv..

[129] Suchowilska E., Wiwart M., Krska R., Kandler W. (2020). Do Triticum aestivum L. and Triticum spelta L. hybrids constitute a promising source material for quality breeding of new wheat varieties?. Agronomy.

[130] Takač V., Tóth V., Rakszegi M., Mikić S., Mirosavljević M., Kondić-Špika A. (2021). Differences in processing quality traits, protein content and composition between spelt and bread wheat genotypes grown under conventional and organic production. Foods.

